# Global, regional, and national burden of tuberculosis due to smoking, 1990-2021: analysis for the Global Burden of Disease study

**DOI:** 10.3389/fimmu.2025.1624090

**Published:** 2025-07-25

**Authors:** Guizeng Zhao, Yating Wu, Chen Song, Yazhou Sun, Shuzhi Zang, Feng Tian, Zhitao Gao, Chenguang Zhang, Xia Wang

**Affiliations:** ^1^ The First Affiliated Hospital of Xinxiang Medical University, Xinxiang, China; ^2^ The Third Affiliated Hospital of Xinxiang Medical University, Xinxiang, China; ^3^ School of Medical Technology, Xinxiang Medical University, Xinxiang, China; ^4^ School of Public Health, Xinxiang Medical University, Xinxiang, China

**Keywords:** tuberculosis, smoking, immune, GBD, 2021

## Abstract

**Background:**

Tuberculosis (TB) is an infectious disease caused by Mycobacterium tuberculosis invading the lungs and other organs, which is a serious threat to human life and health. Recent studies have shown that smoking is an important risk factor for the development of TB and advances the progression of TB through multiple mechanisms that affect the body’s immune function.

**Methods:**

A multidimensional analytical approach was taken to gain a comprehensive understanding of the burden of disease. First, the burden of TB due to smoking (Deaths, DALYs, YLDs, and YLLs) from 1990–2021 was conducted. And then, differences in the burden of disease in 2021 were explored across gender, age, SDI regions, GBD regions and countries. In addition, decomposition analysis was performed to understand the influencing factors of disease burden. Finally, ARIMA and ES models were used to predict trends in disease burden from 2022-2050.

**Results:**

Globally, the number of cases and ASR of TB due to smoking have decreased over time. The burden of disease is heaviest in the middle-aged male population and is much higher than in women. The burden is higher in regions with lower levels of SDI than in those with higher levels of SDI. Australasia has the lowest burden, while India is the country with the highest burden. Projections show a general downward trend in the number of disease burdens from 2022 to 2050, but there is still a need to develop the right strategies to meet the challenges of disease.

**Conclusions:**

Smoking as an independent risk factor for several chronic diseases, this study focuses on the burden of TB due to smoking. Although the results show that the burden situation is decreasing year by year, the state and society still need to increase the publicity of science, raise the awareness of the disease among the public, and develop public health programs to deal with the disease.

## Introduction

Tuberculosis (TB), a persistent infectious disease triggered by Mycobacterium tuberculosis complex pathogens (1), primarily affects pulmonary systems while demonstrating systemic pathogenicity through potential dissemination to hepatic, renal, cerebral, and lymphatic tissues, thereby maintaining its status as a significant global health burden (2). WHO’s 2024 Global TB Report reveals a progressive epidemiological pattern: 2023 recorded 10.8 million incident cases globally, showing a rise from 6.3 million in 2016. Despite a consistent 2% annual incidence reduction observed from 2010-2020, recent surveillance data indicates a concerning 4.6% surge in case rates between 2020 (129/100,000) and 2023 (134/100,000). Notably, TB reemerged as the leading infectious killer in 2023 with 1.25 million fatalities, surpassing HIV/AIDS-related mortality by nearly twofold. Clinical classifications differentiate TB strains by pharmacological responsiveness: drug-sensitive (DS-TB), multidrug-resistant (MDR-TB), and extensively drug-resistant (XDR-TB) variants, with XDR-TB’s therapeutic challenges significantly exacerbating disease morbidity and mortality worldwide (3). MDR-TB represents a heavy burden on the health-care system, with treatment costs 20 times higher than the corresponding costs for DS-TB (3). This evolving resistance profile underscores the critical necessity for developing innovative diagnostic approaches and therapeutic strategies to effectively manage TB pathogenesis and transmission dynamics, thereby advancing global TB control efforts.

More people in the world are infected with Mycobacterium tuberculosis, but only 5-15% will develop TB in their lifetime (4). Biological and environmental factors affect the risk of developing TB in various ways. Biological factors include hormone levels, BMI levels, immune function, lifestyle habits (smoking and alcohol consumption), genetic effects, and other diseases (diabetes mellitus and chronic kidney disease), and immunocompromise in older populations and infants and young children puts them at a high risk for TB (5–11). Environmental factors include exposure and contact with Mycobacterium tuberculosis and smoke exposure (8, 9, 12).

Several studies have reported a causal relationship between smoking and TB, especially with pulmonary TB (13). Not only smokers are at risk of developing TB, but passive smoking, secondhand smoke, and environmental tobacco smoke exposure (ETS) are also contributing factors to active TB (14, 15). A clinical controlled trial showed that smoking delays sputum conversion time, causes attenuated IFN-γ response, and advances disease progression in TB (16). *In vitro* cellular experiments have also shown that smoke exposure reduces effector cytokine responses (IFN-γ, TNF-α, and IL-10) and disrupts containment of mycobacteria by infected human macrophages, which in turn invade the body and cause infection (17). Smoking also causes accumulation of lysosomes, leading to defective or dysfunctional macrophage migration *in vitro*, which can lead to infection (18). All of this available evidence emphasizes that smoking is a major and important risk factor for TB and needs to be better regulated.

As a continuously evolving global epidemiological surveillance system, the Global Burden of Disease (GBD) 2021 repository serves as a foundational resource for epidemiological investigations across diverse disease entities (19), including cancer, blindness, inflammatory bowel disease, schizophrenia, hearing loss and silicosis (20–25). Building upon this research infrastructure, our investigation leveraged GBD 2021 datasets to conduct 1) a longitudinal assessment of smoking-driven TB burden trends, and 2) a stratified comparative analysis of TB subtypes - XDR-TB, DS-TB, and MDR-TB - in relation to smoking.

## Methods

### Data sources

The 2021 iteration of the Global Disease Burden assessment delivers comprehensive modeling of 371 pathological conditions across 204 geopolitical units, spanning 21 GBD regions from 1990 onward. Core datasets are aggregated from multidimensional sources including demographic registries, institutional medical archives, population-based surveillance systems, and nationally representative health surveys (26–28). TB due to smoking—the focus of this investigation—were obtained through systematic retrieval from the standardized repository maintained by the Global Health Data Exchange (GHDx) portal (http://ghdx.healthdata.org/gbd-results-tool). The 95% uncertainty interval of all final estimates of GBD 2021 is generated by the values of the 2.5th and 97.5th percentiles of 500 samplings, and uncertainty is transmitted at every step of the estimation process. This method of calculating the uncertainty interval can reflect the stability and reliability of the results to a certain extent. For regions with missing data, GBD 2021 is simulated using the DisMod-MMR 2.1 model, which extrapolates disease parameters through regional similarity, but the width of 95%UI reflects the estimated fluctuations caused by insufficient data in low-income countries. This study further alleviates this bias through subgroup analysis (29). The data sets used in this study were public, and the study was conducted in accordance with the guidelines for reporting accurate and transparent health estimates (GATHER) (30) and in accordance with the principles of the Declaration of Helsinki (31). Since the GBD 2021 database is public and no identifiable information is included in the analysis, there is no need for ethical approval and informed consent (32).

### Study design

Our longitudinal assessment of smoking-attributable TB burden commenced with quantifying number of cases and age-standardized rates (ASRs) (including Deaths, DALYs, YLDs and YLLs) across the 1990–2021 observation window. Demographic-stratified evaluations dissected burden disparities through multiple lenses: gender, age, developmental status (SDI quintiles), supranational disease clusters (GBD regions), and country-level administrative divisions, prioritizing 2021 cross-sectional patterns. Component decomposition framework precisely apportioned burden variance drivers – demographic aging, population dynamics, and epidemiologic shift coefficients – across the 30-year analytic horizon. The ARIMA model assumes that the autocorrelation of time series is stable over time, which is suitable for linear trend data; the ES model is based on an exponential weighted average, assuming that future trends are similar to historical patterns (33). Burden forecasting models utilizing ARIMA and ES extrapolated smoking-driven TB progression vectors to 2050, with ensemble techniques optimizing prediction robustness. Analytic rigor was ensured through uncertainty simulations (95% UI) with p<0.05 significance thresholds, executed via R statistical computing environment v4.2.2.

## Results

### Temporal trend for GBD of tuberculosis due to smoking from 1990 to 2021

**Figure 1:**
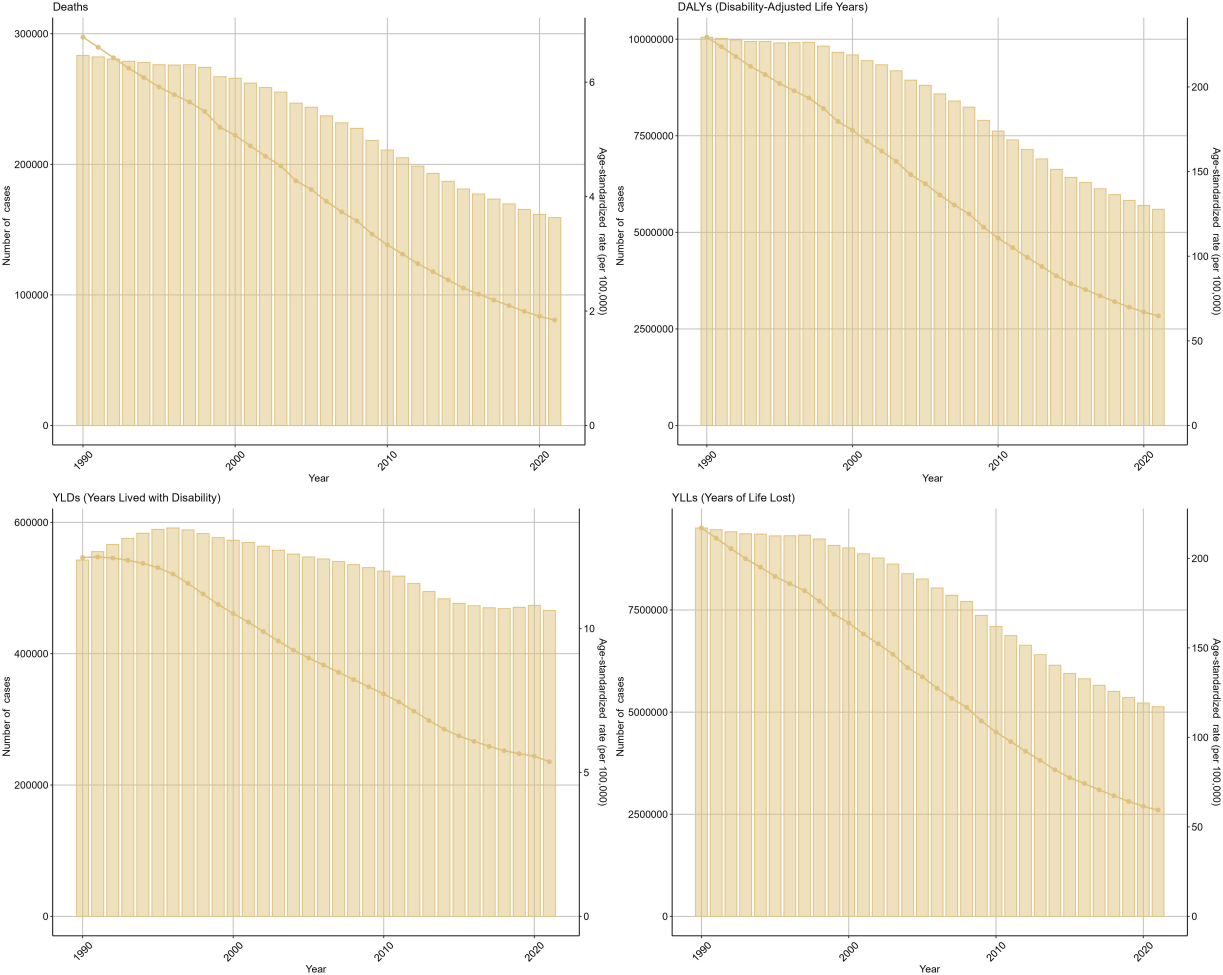
Tuberculosis due to smoking-related GBD of deaths, YLDs, YLLs and DALYs between 1990 and 2021. ASR, age-standardized rate; YLDs, Years Lived with Disability; YLLs, Years of Life Lost; DALYs, disability-adjusted-life-years.

In order to understand the trends in the burden of TB due to smoking from 1990 until 2021, we conducted a multifaceted analysis. From a global perspective, Deaths (EAPC=-3.8, 95%UI: -4.01 to -3.59), DALYs (EAPC=-3.75, 95%UI: -3.94 to -3.57), YLDs (EAPC=-2.51, 95%UI: -2.68 to -2.35), and YLLs (EAPC=- 3.84, 95%UI: -4.03 to -3.65) showed a decreasing trend in ASR values with a similar steepness; in terms of the number of cases, YLDs showed an increasing trend until 1995, after which they have been on a downward slope, while the number of cases for the other three indicators has been on a decreasing trend. Specifically, DALYs declined from 10049593 (95% UI: 7862923-12461356) in 1990 to 5597576 (95% UI: 4372520-6928837) in 2021, and Deaths from 283383 (95% UI: 222230-355234) in 1990 to 159178 (95%UI: 125327-199503), YLDs from 542586 (95%UI: 343280-791616) to 465841 (95%UI: 293897-680068), and YLLs from 9507007 (95%UI: 7468190-11885793) to 5131735 (95%UI: 4006172-6393632). The results for the other three subcategories of TB due to smoking showed that the trend for drug-sensitive TB due to smoking was the same as that for the broader category, whereas both the number of cases and the ASR for drug-resistant TB due to smoking first increased and then decreased over time from very low values, suggesting that the development of drug resistance is the main reason why the burden of TB due to smoking remains high (**Figure 1**, **Supplementary Figure S1**, **Table 1**).

**Table 1:** The change of tuberculosis due to smoking-related GBD of Deaths, YLDs, YLLs and DALYs globally between 1990 and 2021.

Type	Measure	Number in 1990	ASR in 1990	Number in 2021	ASR in 2021	EAPC
TB due to smoking	DALYs	10049593 (7862923-12461356)	229.42 (179.31-284.8)	5597576 (4372520-6928837)	64.79 (50.61-80.14)	-3.75 (-3.94--3.57)
	Deaths	283383 (222230-355234)	6.79 (5.31-8.52)	159178 (125327-199503)	1.84 (1.45-2.31)	-3.8 (-4.01--3.59)
	YLDs	542586 (343280-791616)	12.47 (7.88-18.23)	465841 (293897-680068)	5.38 (3.38-7.85)	-2.51 (-2.68--2.35)
	YLLs	9507007 (7468190-11885793)	216.95 (170.41-271.4)	5131735 (4006172-6393632)	59.41 (46.39-74.03)	-3.84 (-4.03--3.65)
extensive drug-resistant TB due to smoking	DALYs	0 (0-0)	0 (0-0)	54601 (25693-99898)	0.63 (0.3-1.16)	11.22 (6.86-15.75)
	Deaths	0 (0-0)	0 (0-0)	1565 (705-2925)	0.02 (0.01-0.03)	-2.46 (-5.91-1.13)
	YLDs	0 (0-0)	0 (0-0)	1691 (919-3205)	0.02 (0.01-0.04)	5.3 (2.15-8.55)
	YLLs	0 (0-0)	0 (0-0)	52910 (24357-98303)	0.61 (0.28-1.14)	3.32 (1.48-5.19)
drug-sensitive TB due to smoking	DALYs	9930532 (7776023-12278437)	226.68 (177.36-280.58)	5043552 (3933603-6275141)	58.37 (45.51-72.68)	-4 (-4.16--3.85)
	Deaths	279795 (219080-351659)	6.7 (5.24-8.43)	143003 (110656-180190)	1.66 (1.28-2.09)	-4.05 (-4.23--3.86)
	YLDs	538173 (340939-786319)	12.37 (7.82-18.07)	441549 (278952-639736)	5.1 (3.21-7.4)	-2.61 (-2.76--2.45)
	YLLs	9392359 (7384313-11732655)	214.31 (168.41-268.22)	4602003 (3573802-5746206)	53.28 (41.38-66.53)	-4.11 (-4.26--3.95)
multidrug-resistant TB due to smoking	DALYs	119061 (40947-300673)	2.74 (0.94-6.92)	499422 (194864-1030642)	5.78 (2.26-11.93)	0.07 (-1.04-1.19)
	Deaths	3589 (1230-9173)	0.09 (0.03-0.22)	14610 (5350-30600)	0.17 (0.06-0.35)	-0.06 (-1.13-1.01)
	YLDs	4413 (1657-9962)	0.1 (0.04-0.23)	22601 (10914-42734)	0.26 (0.13-0.49)	0.14 (-0.82-1.12)
	YLLs	114648 (39268-292523)	2.64 (0.9-6.74)	476821 (182508-978954)	5.52 (2.11-11.33)	0.06 (-1.05-1.19)

ASR, age-standardized rate; YLDs, Years Lived with Disability; YLLs, Years of Life Lost; DALYs, disability-adjusted-life-years.

**Figure 2:**
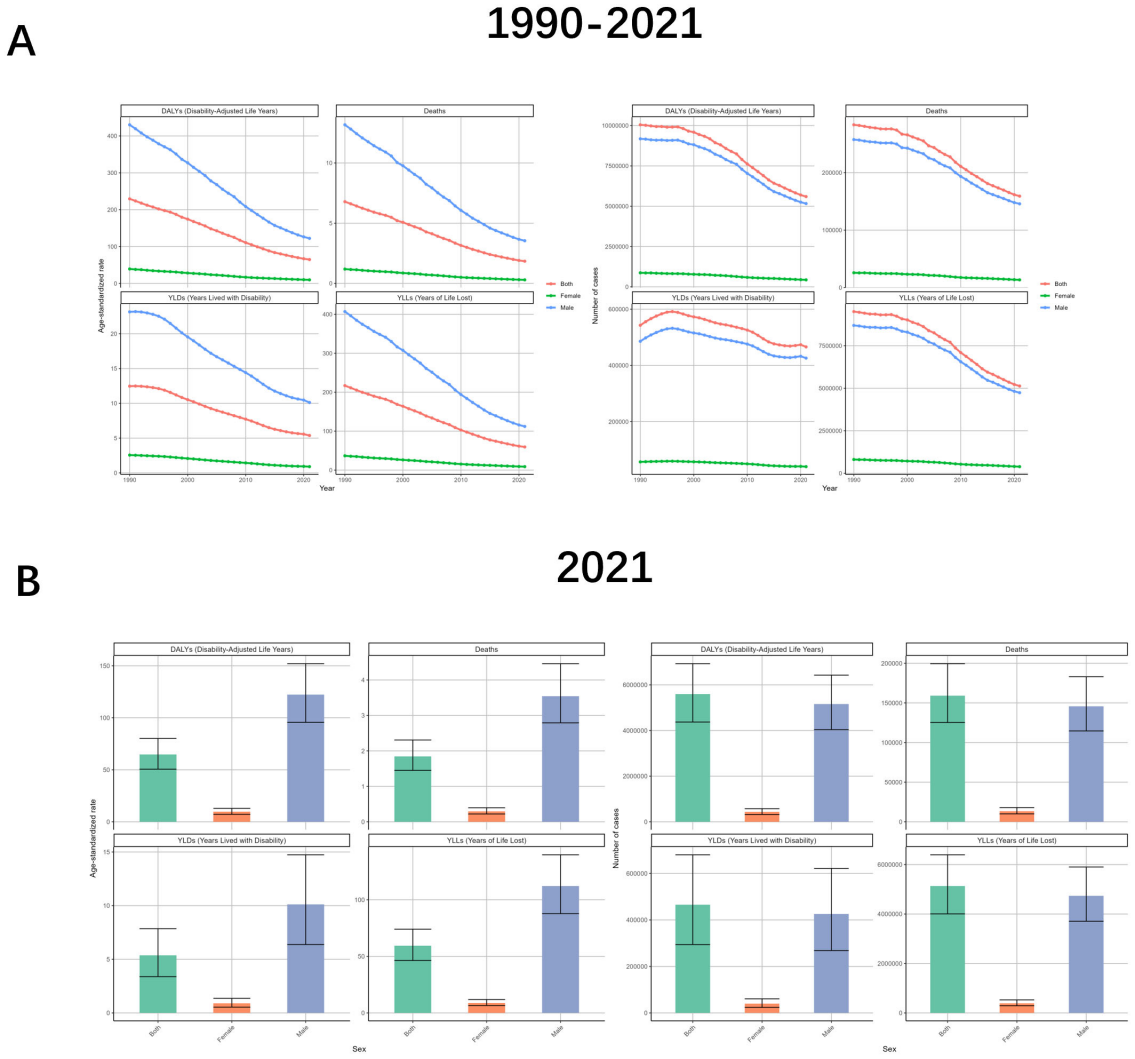
Tuberculosis due to smoking-related GBD of deaths, YLDs, YLLs and DALYs between for different genders between 1990 and 2021. **(A)** Temporal trends in DALYs, deaths, YLDs, and YLLs for both sexes combined, females, and males, 1990-2021. **(B)** Sex-specific comparisons of DALYs, deaths, YLDs, and YLLs in 2021. ASR, age-standardized rate; YLDs, Years Lived with Disability; YLLs, Years of Life Lost; DALYs, disability-adjusted-life-years.

**Figure 3:**
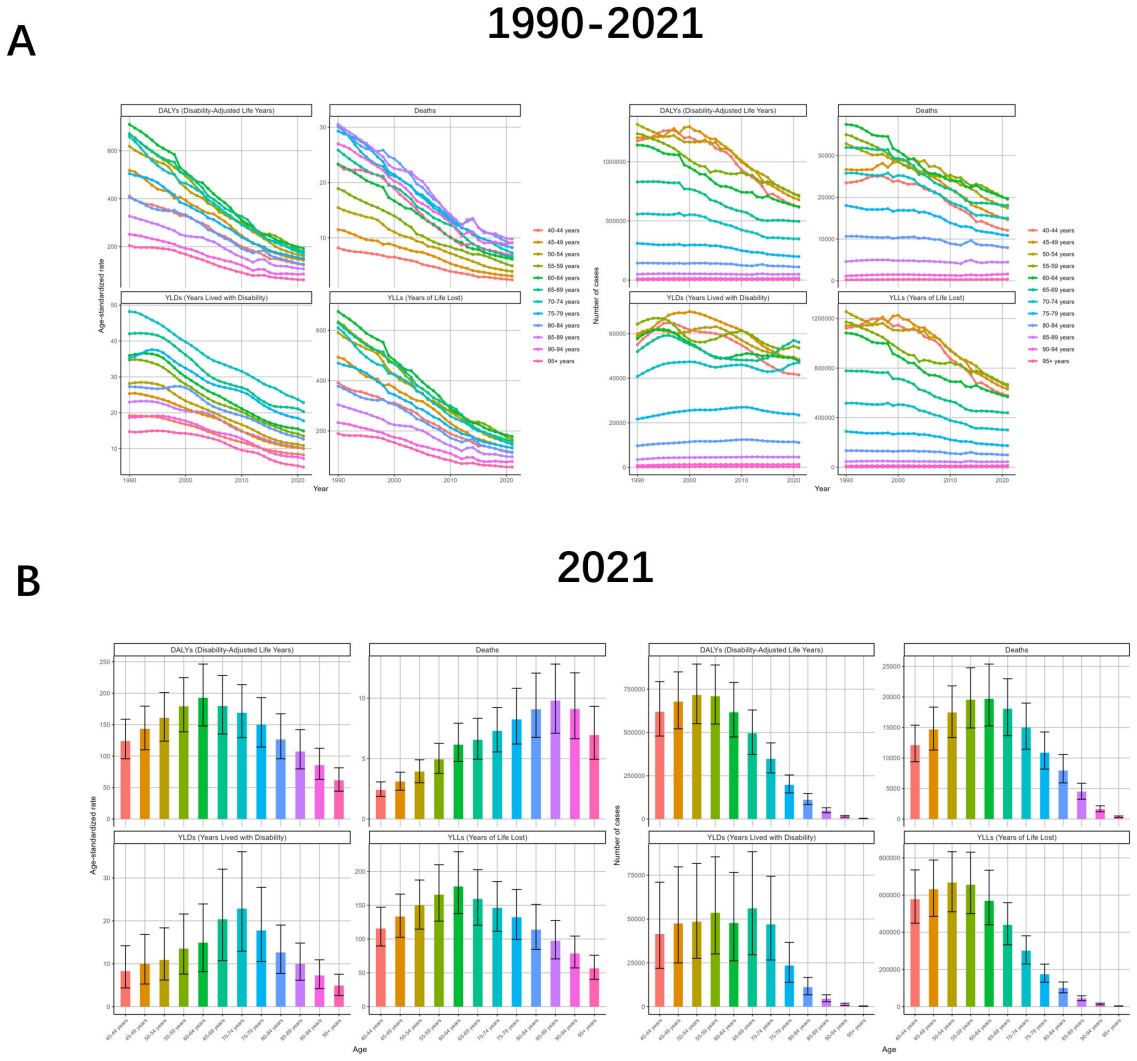
Tuberculosis due to smoking-related GBD of deaths, YLDs, YLLs and DALYs between for different ages between 1990 and 2021. **(A)** Temporal patterns of DALYs, deaths, YLDs, and YLLs across age groups, 1990-2021. **(B)** Age-specific distributions of disease burden metrics in 2021. ASR, age-standardized rate; YLDs, Years Lived with Disability; YLLs, Years of Life Lost; DALYs, disability-adjusted-life-years.

Analyze the burden differently by gender. From the data, the burden profile for both males and females is consistent with the global trend, but the burden is much higher for males than for females, and the change curve for females is all almost close to the abscissa. drug-sensitive or drug-resistant TB due to smoking is also consistent, and all follow the trend of being much higher for males than for females. Analyzing by age, the trend of ASR change in all age groups is almost the same as the global one, but there is a small fluctuation in the age 80–94 in 2012-2014; and in the number of cases, the fluctuation in the group older than 75 years is smaller but declining in general, while the rest of the age groups have an upward movement in the early years and then downward (**Figures 2**, **3**, **Supplementary Figures S2, 3**, **Supplementary Tables S1, 2**).

**Figure 4:**
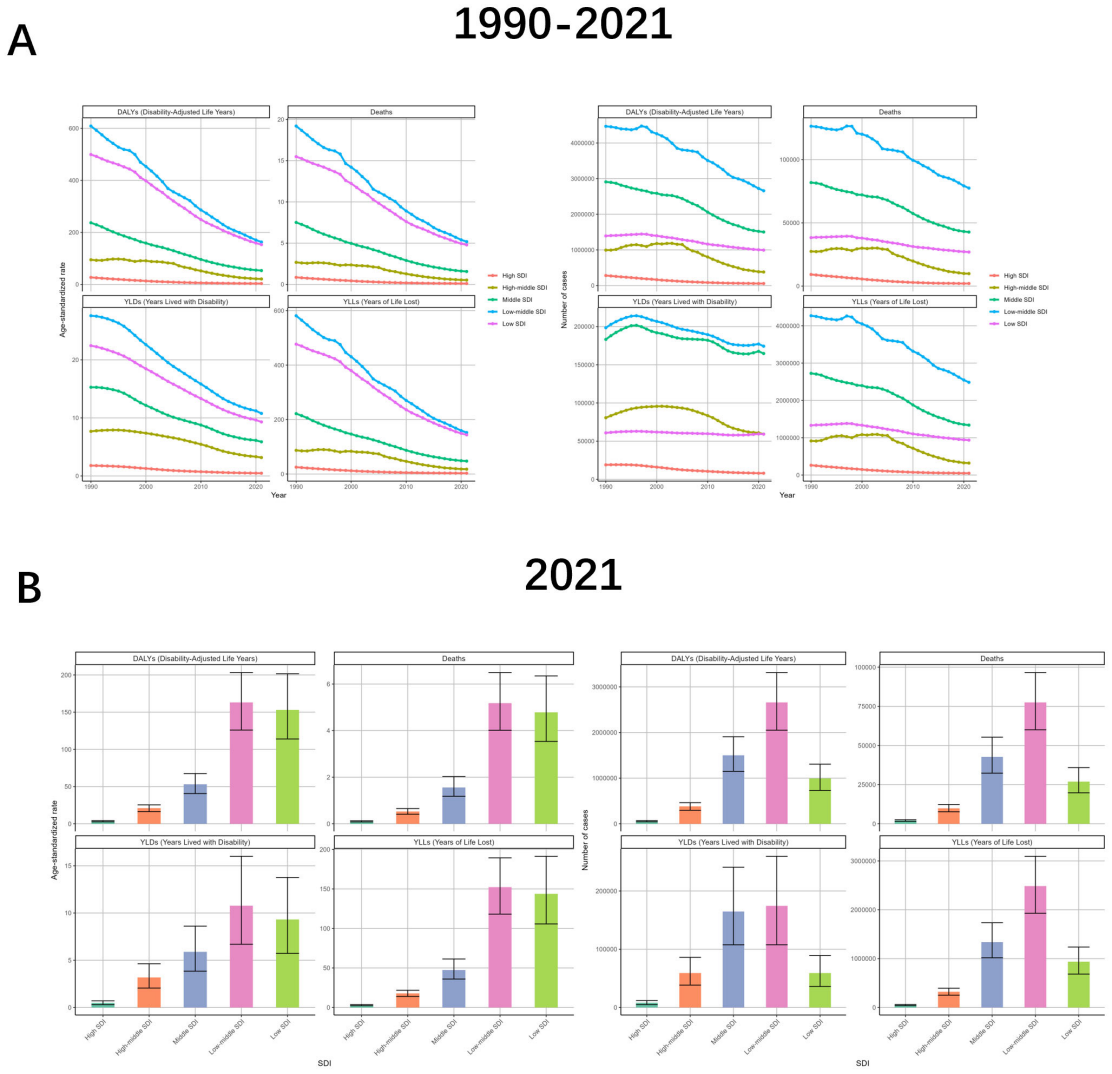
Tuberculosis due to smoking-related GBD of deaths, YLDs, YLLs and DALYs between for different SDI regions between 1990 and 2021. **(A)** Line graphs of 1990-2021 trends in DALYs, deaths, YLDs, and YLLs across Socio-Demographic Index (SDI) levels. **(B)** 2021 SDI-level comparisons of disease burden metrics through bar charts. ASR, age-standardized rate; YLDs, Years Lived with Disability; YLLs, Years of Life Lost; DALYs, disability-adjusted-life-years.

The burden of TB due to smoking varies significantly depending on the level of SDI. The curve of change in ASR shows that the lower the level of SDI, the greater the absolute value of the slope of the downward curve is likely to be. This is reflected in the fact that Low-middle SDI>Low SDI>Middle SDI>High-middle SDI>High SDI, with the ASR of the High SDI region infinitely close to 0. The number of cases is similar to the change of ASR, but the value of High-middle SDI is almost unchanged before 2005. As for the drug-resistant TB, the trend in the High-middle SDI region is the closest to the global one, and the trend in the High SDI region seldom coincides with the global change curve (**Figure 4**, **Supplementary Figure S4**, **Supplementary Table S3**).

**Figure 5:**
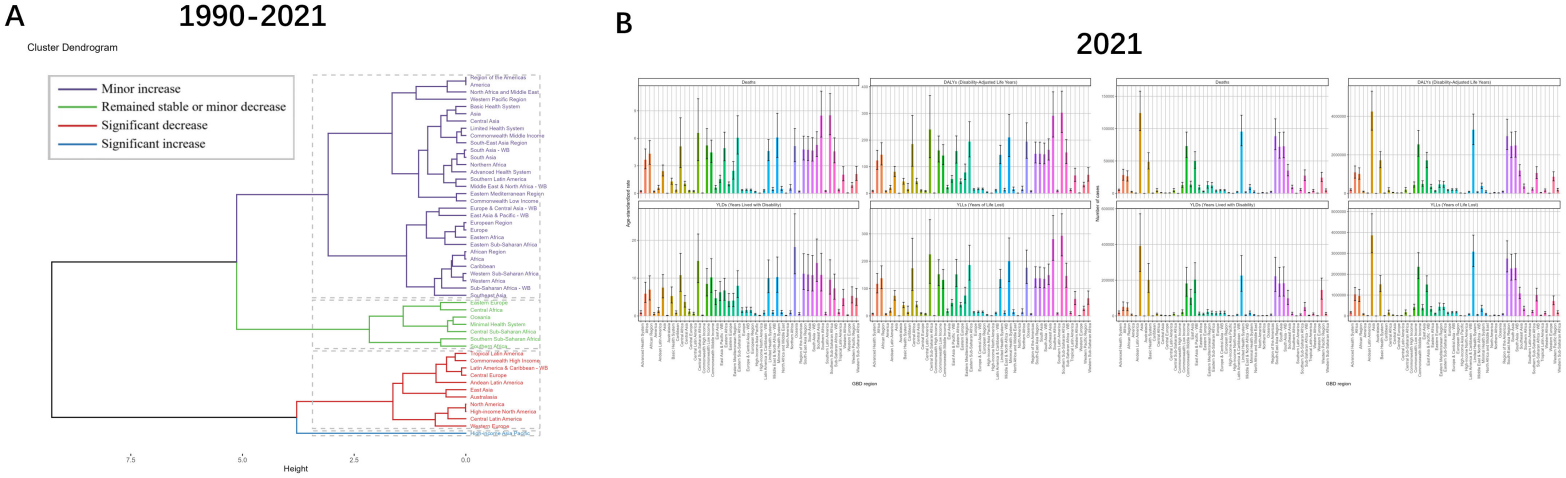
Results of cluster analysis based on the EAPC values of tuberculosis due to smoking-related age-standardized rates for deaths and DALYs from 1990 to 2021 and GBD in’2021. **(A)** Dendrogram of 1990-2021 trends with color-coded classifications: minor increase (violet), stable/minor decrease (green), significant decrease (red), significant increase (blue). **(B)** 2021 multi-category data visualization through rainbow-colored bar charts. EAPC, estimated annual percentage change; DALYs, disability-adjusted-life-years.

In order to better reflect changes in burden across GBD regions, a hierarchical cluster analysis was conducted by the combined ASRs. TB due to smoking, there were 11 regions with significant decrease in burden: Tropical Latin America, Commonwealth High Income, Latin America & Caribbean - WB, Central Europe, Andean Latin America, East Asia, Australasia, North America, High-income North America, Central Latin America, Western Europe. In contrast, the only region with a significant increase in burden was High-income Asia Pacific. The regional changes in TB due to smoking for the remaining three classifications are shown in **Supplementary Figure S5** (**Figure 5**, **Supplementary Figures S5**, **Supplementary Table S4**).

**Figure 6:**
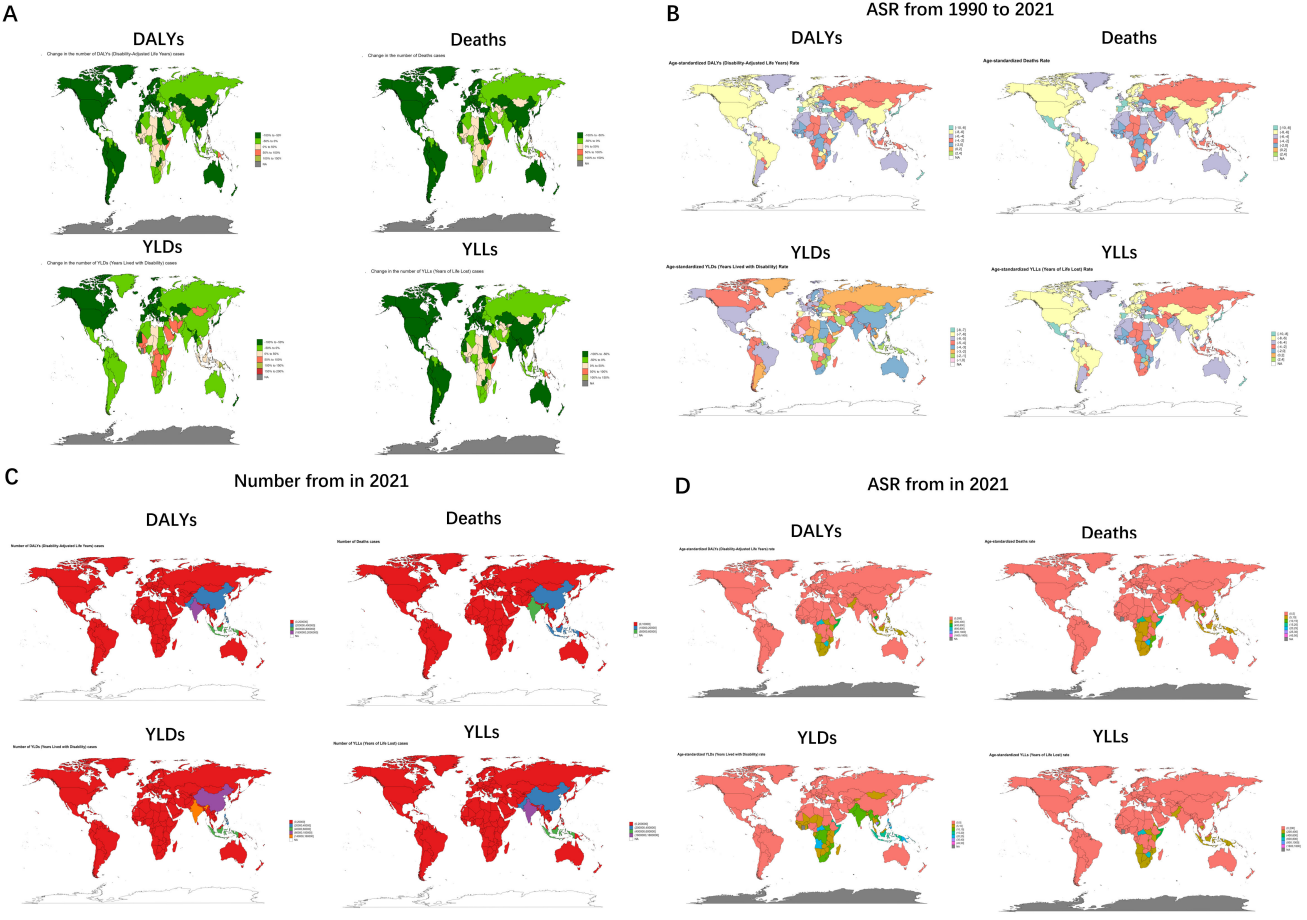
Tuberculosis due to smoking-related numbers and ASRs of deaths, YLDs, YLLs and DALYs between for different countries between 1990 and 2021. **(A)** Global 1990-2021 DALYs, deaths, YLDs, and YLLs distribution via green gradient maps. **(B)** Age-standardized rate variations through multi-colored mapping. **(C)** 2021 numerical comparisons in red-blue color spectrum. **(D)** 2021 age-standardized rates with corresponding color scheme. ASR, age-standardized rate; YLDs, Years Lived with Disability; YLLs, Years of Life Lost; DALYs, disability-adjusted-life-years.

The change in the burden of the same disease varies among administrative countries according to social development and policy responses, and the countries with the highest increase in burden for DALYs, Deaths and YLLs are Lesotho, with EAPC values of 2.73 (95% UI: 2.09 to 3.38), 2.4 (95% UI: 1.7 to 3.1) and 2.78 (95% UI: 2.14-3.43), whereas the change in burden for YLDs were all negative, all showing a downward trend, the Marshall Islands (EAPC = -0.26, 95%: -0.88 to 0.35) showed the smallest degree of decline. The country with the largest reduction in burden among DALYs, Deaths, and YLDs was Hungary, with EAPC values of -8.66 (95%UI: -9.12 to -8.21), -8.89 (95% UI: -9.41 to -8.37) and -6.45 (95% UI: -6.86 to -6.03), while New Zealand had the largest decrease in YLLs at -9.03 (95% UI: -9.66 to -8.39) (**Figure 6**, **Supplementary Figure S6**, **Supplementary Table S5**).

### The disease burden of tuberculosis due to smoking in 2021

In order to provide a more detailed picture of the recent burden of disease for TB due to smoking, we have described the disease in detail for 2021, which will be analyzed stratified by different subtypes.

Gender-induced differences in lifestyle habits and physiology result in serious differences in the burden of TB due to smoking. The number of cases in males is almost 10–12 times higher than that of females, with DALYs of 5164516 (95% UI: 4038185-6427927), Deaths of 145795 (95% UI: 114686-183032), YLDs of 425908 (95% UI: 268314-621577), YLLs of 4738608 (95% UI: 3707451-5901668). And males were also as much as 11-12.5 times more likely than females to have an ASR. And the TB results were similar for the other three subcategories (**Figure 2**, **Supplementary Figure S6**, **Supplementary Table S1**).

When analyzed in terms of age, the ASRs of all four indicators showed an increasing and then decreasing trend with age, but the age strata in which the maximum values were located were almost always different. DALYs (192.8, 95%UI: 148.09-246.33), Deaths (9.8, 95%UI: 7.1-12.82), YLDs (22.83, 95%UI. 12.93-36.13), and YLLs (177.87, 95%UI: 137.63-229.31) were localized in the age groups of 60-64, 85-89, 70–74 and 60-64, respectively. The number of cases also showed an increasing and then decreasing trend with age, with DALYs (716324, 95%UI: 550562-894833) and YLLs (667893, 95%UI: 510153-833665) being the largest in the 50–54 age group, Deaths (19690, 95%UI: 15234-25384) were the largest at 60-64, YLDs (56152, 95%UI: 29605-88418) were the largest at 65-69 (**Figure 3**, **Supplementary Figure S7**, **Supplementary Table S2**).

The burden varied markedly by SDI level. The horizontal ASRs were low in the High SDI, High-middle SDI, and Middle SDI regions, while the values were high in the other two SDI regions, and were especially highest in the Low-middle SDI region, with DALYs, Deaths, YLDs, and YLLs of 162.98 (95% UI: 125.96-203.17), 5.18 (95% UI: 4.01-6.49), 10.77 (95% UI: 6.69-16.01) and 152.21 (95% UI: 118.1-189.33) respectively. In terms of the number of cases, Low-middle SDI still occupies the largest value, followed by Middle SDI regions, while High SDI and High-middle SDI regions continue to have small values. The relationship between the burden of drug-sensitive TB or multidrug-resistant TB due to smoking and SDI was consistent with the broad categories. However, the case of extensive drug-resistant TB due to smoking was more specific, with the High-middle SDI region being at the highest point in terms of both ASR and number of cases (**Figure 4**, **Supplementary Figure S8**, **Supplementary Table S3**).

The burden among GBDs varied depending on a number of factors. The maximum ASRs for Deaths, DALYs and YLLs were all in Southern Sub-Saharan Africa, at 302.75 (95% UI: 228.45-383.61), 8.51 (95% UI: 6.38-10.87) and 293.17 (95%UI: 219.3-373.23), while YLDs were localized in Oceania (18.18, 95%UI: 11.11-26.99). Asia, on the other hand, bagged the maximum number of cases for DALYs, Deaths, YLDs and YLLs with 4248739 (95%UI: 3281978-5307890), 124081 (95%UI: 96903-157939), 391500 (95%UI: 249805-571994) and 3857239 (95% UI: 3023363-4893719). Australasia had the smallest burden with 225 (95%UI: 168-289), 7 (95%UI: 6-10), 43 (95%UI: 24-67), and 183 (95%UI: 139-234) cases of DALYs, Deaths, YLDs, and YLLs, respectively, and ASRs of 0.51 (95%UI: 0.38-0.65), 0.01 (95%UI: 0.01-0.02), 0.1 (95%UI: 0.06-0.16) and 0.4 (95%UI: 0.3-0.51), respectively (**Figure 5**, **Supplementary Figure S9**, **Supplementary Table S4**).

Burden varied significantly between countries, with several countries with case counts as low as 0, including but not limited to American Samoa, Andorra, Antigua and Barbuda, Barbados, Bermuda, Cook Islands, Dominica, and San Marino. while India’s burden numbers all rode high, with values of 1924894 (95% UI: 1414529-2640679), 56395 (95% UI: 41672-78399), 147732 (95% UI: 88598-221286), and 1777161 (95%UI: 1307168-2492317) for its DALYs, Deaths, YLDs, and YLLs, respectively. While at the level of ASR, Bermuda and Andorra had the smallest, Kiribati (46.14, 95%UI: 30.07-66.69) had the largest value of YLDs, while DALYs (1634.72, 95%UI: 953.9-2296.09), Deaths (46.23, 95%UI: 27.14 -65.3) and YLLs (1621.69, 95%UI: 942.98-2276.25) all had maximum values for Lesotho (**Figure 6**, **Supplementary Table S5**).

### Drivers of tuberculosis due to smoking epidemiology – aging, population, and epidemiological change

To gain insight into the factors that contribute to TB due to smoking, we used decomposition analyses to explore the effects of aging, population, and epidemiological change in the burden associated with different sexes and different SDI levels.

**Figure 7:**
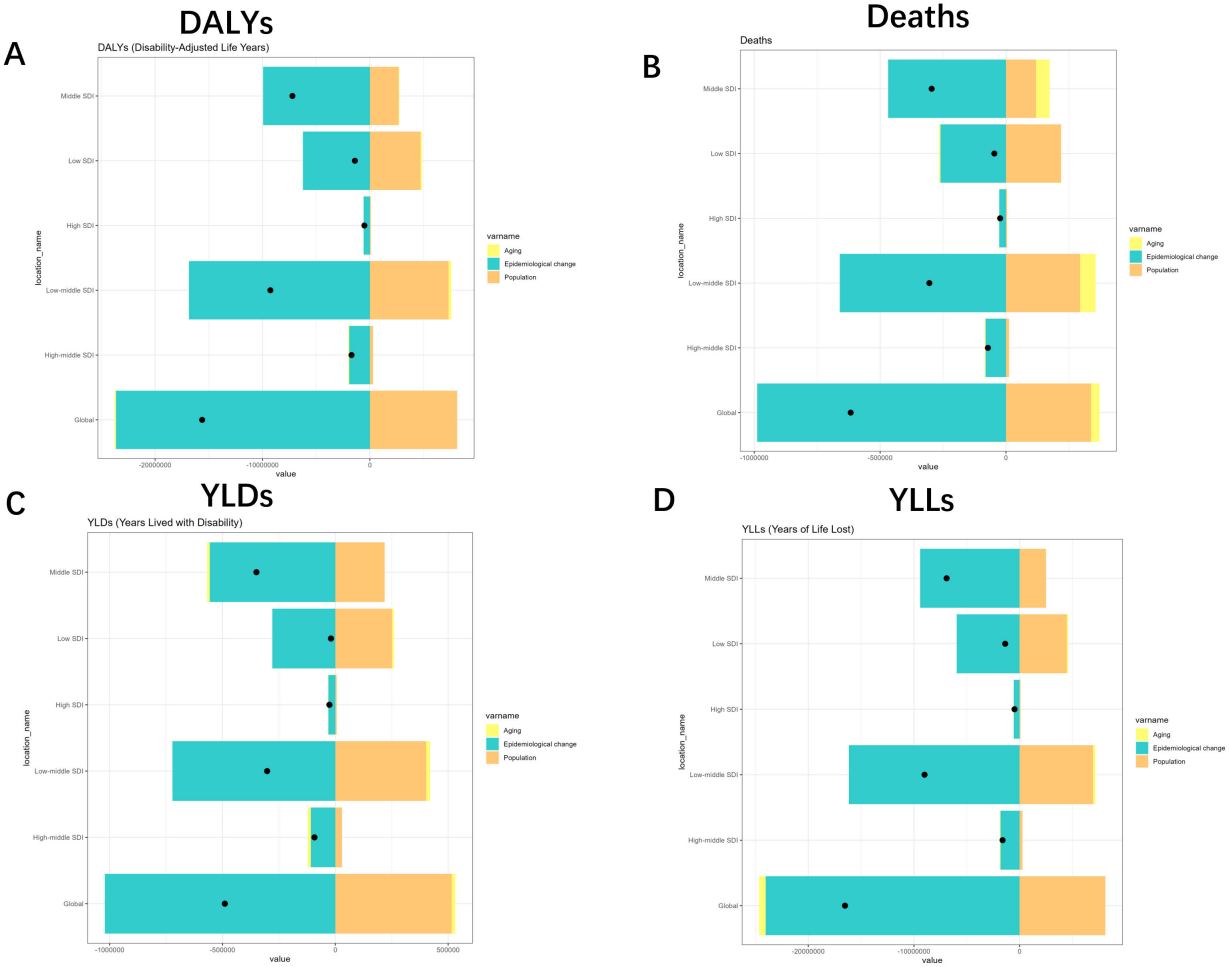
Changes in Deaths, DALYs, YLDs and YLLs for Tuberculosis due to smoking according to population-level determinants of ageing, population growth, and epidemiological change from 1990 to 2021 for different SDI. **(A)** SDI-quintile comparison of DALYs with turquoise (aging), yellow (epidemiological change), and orange (population) indicators. **(B)** Mortality patterns across SDI quintiles with identical color coding. **(C)** YLDs distribution by SDI quintile and demographic factors. **(D)** YLLs variations across population characteristics. YLDs, Years Lived with Disability; YLLs, Years of Life Lost; DALYs, disability-adjusted-life-years.

**Figure 8:**
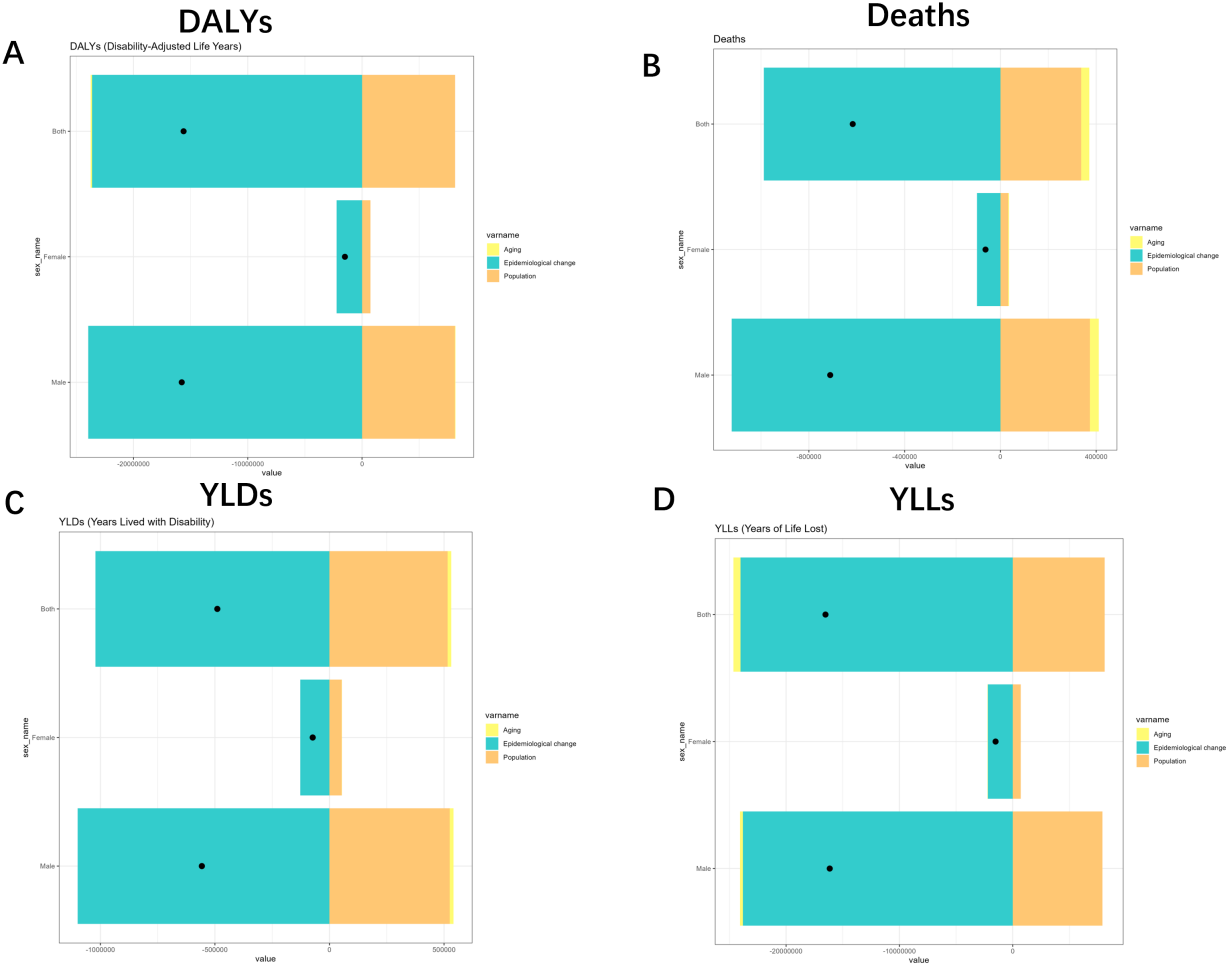
Changes in Deaths, DALYs, YLDs and YLLs for Tuberculosis due to smoking according to population-level determinants of ageing, population growth, and epidemiological change from 1990 to 2021 for different gender. **(A)** DALYs analysis through tri-color segmented bars with data markers. **(B)** Deaths metrics visualization with identical structural format. **(C)** YLDs representation maintaining consistent visual framework. **(D)** YLLs comparisons using unified graphical approach. YLDs, Years Lived with Disability; YLLs, Years of Life Lost; DALYs, disability-adjusted-life-years.

Specifically, population all caused a reduction in the burden of DALYs (-52.08%), Deaths (-54.74%), YLDs (-105.25%), and YLLs (-49.12%), whereas Epidemiological Changes all caused an increase in DALYs, Deaths, YLDs, and YLLs of 151.3%, 160.19%, 208.58% and 145.41%. Aging, on the other hand, caused an increase in DALYs and YLLs by 0.77% and 3.71%, respectively, and a decrease in Deaths and YLDs by 5.46% and 3.33%, respectively. The effects on different SDIs and different sexes are shown in **Figures 7**, **8**. Overall, aging and population caused almost a decrease in burden, while epidemiologic factors caused an increase in burden (**Figures 7**, **8**, **Supplementary Figures S11, 12**).

### The predicted results of disease burden for tuberculosis due to smoking from 2022 to 2050

**Figure 9:**
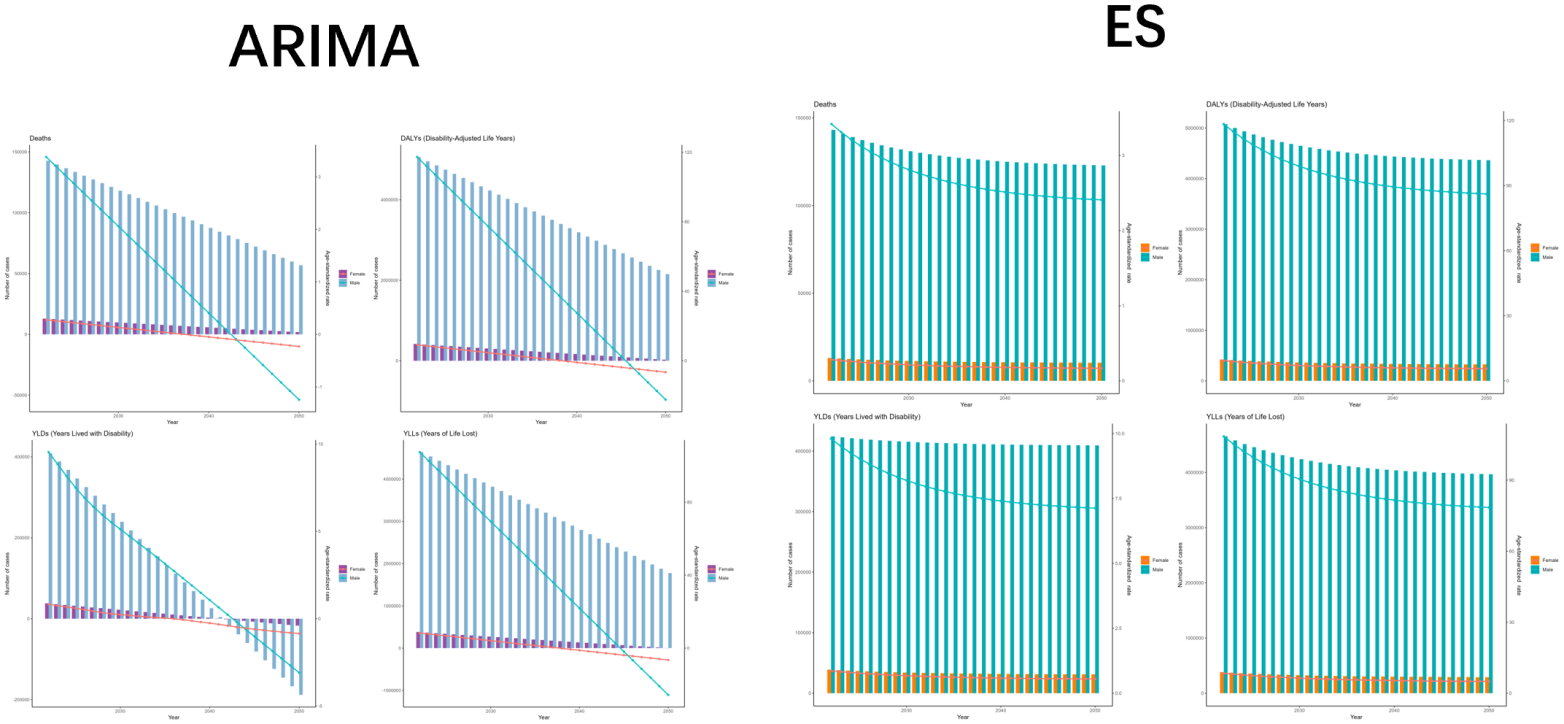
The predicted results in Tuberculosis due to smoking-related GBD of deaths, YLDs, YLLs and DALYs from 2022 to 2050 by ARIMA and ES model. **(A)** ARIMA model projections for deaths, QALYs, and YLLs with trend indicators. **(B)** ES model forecasts of identical metrics showing consistent patternss. ASR, age-standardized rate; YLDs, Years Lived with Disability; YLLs, Years of Life Lost; DALYs, disability-adjusted-life-years.

In order to better develop prevention and treatment measures to intervene in the development of TB due to smoking, we used ARIMA and ES models to predict the burden in the coming decades. Surprisingly, the results of both models showed a decreasing trend in the number of cases and ASR of TB due to smoking, with the difference being the different slopes of the decreasing curves. This is a promising result. In contrast, the results of other subcategories of TB showed a decline, except for MDR-TB for which the ARIMA results showed an increase in burden. Although the results show a promising trend, there is still a need to focus on the development of TB due to smoking (**Figure 9**, **Supplementary Figure S12**).

## Discussion

To gain a comprehensive and in-depth understanding of the current status of the burden of TB due to smoking, we analyzed Deaths, DALYs, YLDs, and YLLs from TB due to smoking over the period 1990-2021, with subcategories of DS-TB, MDR-TB, and XDR-TB. Overall, the number of cases and ASR of TB due to smoking showed a decreasing trend, but there was an increase in the burden of resistant TB, suggesting the need to focus on the incidence of resistant TB to curb further deterioration in a timely manner.

Our results showed that the burden of TB due to smoking was significantly higher in male than in female. This may be attributed to two main reasons (34, 35): 1) biologic factors: hormonal differences between males and females, and the influence of female hormones on immune function, which cause stronger protection against external cytotoxic viral invasions; and 2) abiotic factors: males are more susceptible to bad habits such as smoking, and males’ willingness to diagnose and treat diseases is generally lower than that of females. The impact of age on the burden of disease also deserves to be explored in depth. As can be seen from the results, the burden of disease is relatively higher in all age groups 50-69. With age, the immune system function declines and becomes more susceptible to Mycobacterium tuberculosis, or Mycobacterium tuberculosis latently carried in early life becomes active (36). Secondly, smoking, as a risk factor for chronic obstructive pulmonary disease (COPD) and diabetes mellitus (DM), increases the risk of developing these chronic diseases in middle-aged and older age groups, and the combined presence of these chronic diseases further reduces the immune function of the body and increases the risk of TB development (37). Studies have pointed out that smoking impairs the autophagy function of macrophages, and autophagy is essential for the removal of Mycobacterium tuberculosis. Autophagy defects caused by smoking may make it more difficult for macrophages to resist Mycobacterium tuberculosis infection, thereby increasing the risk of tuberculosis (38).

There are large differences in economic levels between regions with different SDI levels, leading to huge differences in the burden of TB due to smoking. A significant correlation between economic level and smoking prevalence has been reported, with lower-income populations typically having higher smoking prevalence than higher-income populations, as is the case in the United States (39). However, there is also evidence that a decline in income causes an increase in smoking cessation rates (40). Combined with the results of this study, the burden of TB due to smoking is generally lower in high SDI areas than in low SDI areas, which may be related to the degree of public health wellness science, and the masses in high SDI areas have stronger health awareness. However, the burden of extensively drug-resistant TB was also higher in higher level SDI areas, which suggests that drug-resistant TB is still a major threat to the public health system at present. Although the high-income Asia-Pacific region is a high SDI region, the tobacco tax rate is significantly lower than that of Australasia, resulting in a slow decline in smoking rates, thereby maintaining the ‘ abnormal ‘ upward trend of TB burden (41).

When examining geographical disparities in disease prevalence, Australasia demonstrates the lowest regional health burden—a finding that aligns with prior research. This favorable outcome stems from multiple interrelated factors: socioeconomic advancement, well-established healthcare infrastructure, and effective public health initiatives. Furthermore, these nations implement enhanced TB management strategies through comprehensive monitoring systems and preventive measures like immunization programs and community health education. Their capacity for rapid case identification and therapeutic intervention significantly curbs disease spread and improves treatment outcomes, thereby minimizing both infection rates and TB-related fatalities (42, 43).

India is riding high on the burden of TB due to smoking, which can be attributed to these factors. First, India is the second largest country in terms of tobacco use and has a “dual epidemic” of smoking and TB, with smoking weakening the immune system and increasing the risk of TB infection (15, 44). One is the penetration of tobacco industry marketing. The coverage of tobacco advertising in rural areas is 68%, and the accessibility of cheap tobacco is high. Secondly, the TB diagnosis and treatment system is weak, and the misdiagnosis rate of private clinics is 41%, resulting in a median delay of TB diagnosis in smokers of 28 days (45). Secondly, the low level of economic development and the increased economic burden can increase the incidence of malnutrition, which increases susceptibility to TB (46, 47). Thirdly, the backwardness of health-care systems and policies is such that the public’s perception of the disease largely influences the diagnosis and treatment of the disease, and incomplete intervention policies can lead to a more severe disease burden (48, 49).

Although this study utilizes GBD data to provide a detailed assessment of the burden of TB due to smoking, which provides an important basis for the situation of relevant TB at the global, regional, and national levels, it also suffers from a number of limitations that are common to GBD studies (50–52). 1) There are data collection breaks in the disease surveillance systems of countries with low levels of economic development, with underreporting rates of up to 30-60%, which may mask region-specific epidemiological characteristics.2) There is a response time lag between the annual update cycle of GBD and public health emergencies, and there may be a lag of 3–5 years.3) There are limitations in the model architecture, and although the Bayesian meta-regression framework is able to integrate data from multiple sources, the *a priori* distribution setting is still affected by the subjective judgment of the researcher. At the same time, in view of the limitations of the current model for MDR-TB prediction, subsequent studies will focus on incorporating real-time drug resistance monitoring data and developing Bayesian model integration mechanism parameters to improve the robustness of prediction.

## Conclusions

Our results show that TB due to smoking has the greatest impact on men in the peak economic productivity age group, posing a significant risk to global health systems. The results suggest that both smoking-related TB cases and ASRs have declined in recent years and that the burden continues to trend downward in subsequent decades. While this changing trend is encouraging, rational policies are still needed to reduce TB morbidity and mortality. It is suggested that relevant public health departments and hospitals can embed smoking cessation clinics in TB diagnosis and treatment institutions, and adopt the dual-track model of ‘ TB treatment + smoking cessation intervention ‘. At the same time, the popular science content related to smoking and TB can be pushed through mobile APP to improve patient compliance. In addition, TB screening can be included in the annual physical examination program among smokers aged 50–69 years.

## Data Availability

The original contributions presented in the study are included in the article/**Supplementary Material**. Further inquiries can be directed to the corresponding authors.
