# Supplementary material for: Global, regional, and national burden of tuberculosis due to smoking, 1990-2021: analysis for the Global Burden of Disease study
Source: Front Immunol. 2025 Jul 25;16:1624090. doi: 10.3389/fimmu.2025.1624090 (PMC12331698; doi:10.3389/fimmu.2025.1624090)

**Table legend**

**Table S1.** Tuberculosis due to smoking-related GBD of deaths, YLDs, YLLs and DALYs for different gender between 1990 and 2021. Abbreviations: ASR, age-standardized rate; YLDs, Years Lived with Disability; YLLs, Years of Life Lost; DALYs, disability-adjusted-life-years.

**Table S2.** Tuberculosis due to smoking-related GBD of deaths, YLDs, YLLs and DALYs for different age groups between 1990 and 2021. Abbreviations: ASR, age-standardized rate; YLDs, Years Lived with Disability; YLLs, Years of Life Lost; DALYs, disability-adjusted-life-years.

**Table S3.** Tuberculosis due to smoking-related GBD of deaths, YLDs, YLLs and DALYs for different SDI regions between 1990 and 2021. Abbreviations: ASR, age-standardized rate; YLDs, Years Lived with Disability; YLLs, Years of Life Lost; DALYs, disability-adjusted-life-years.

**Table S4.** Tuberculosis due to smoking-related GBD of deaths, YLDs, YLLs and DALYs for different GBD regions between 1990 and 2021. Abbreviations: ASR, age-standardized rate; YLDs, Years Lived with Disability; YLLs, Years of Life Lost; DALYs, disability-adjusted-life-years.

**Table S5.** Tuberculosis due to smoking-related GBD of deaths, YLDs, YLLs and DALYs for different countries between 1990 and 2021. Abbreviations: ASR, age-standardized rate; YLDs, Years Lived with Disability; YLLs, Years of Life Lost; DALYs, disability-adjusted-life-years.

**Figure legend**

**Figure S1.** Three types of tuberculosis due to smoking-related GBD of deaths, YLDs, YLLs and DALYs between 1990 and 2021. Abbreviations: ASR, age-standardized rate; YLDs, Years Lived with Disability; YLLs, Years of Life Lost; DALYs, disability-adjusted-life-years.

**Figure S2.** Three types of tuberculosis due to smoking-related GBD of deaths, YLDs, YLLs and DALYs between for different gender between 1990 and 2021. Abbreviations: ASR, age-standardized rate; YLDs, Years Lived with Disability; YLLs, Years of Life Lost; DALYs, disability-adjusted-life-years.

**Figure S3.** Three types of tuberculosis due to smokin-related GBD of deaths, YLDs, YLLs and DALYs between for different ages between 1990 and 2021. Abbreviations: ASR, age-standardized rate; YLDs, Years Lived with Disability; YLLs, Years of Life Lost; DALYs, disability-adjusted-life-years.

**Figure S4.** Three types of tuberculosis due to smoking-related GBD of deaths, YLDs, YLLs and DALYs between for different SDI regions between 1990 and 2021. Abbreviations: ASR, age-standardized rate; YLDs, Years Lived with Disability; YLLs, Years of Life Lost; DALYs, disability-adjusted-life-years.

**Figure S5.** Results of cluster analysis based on the EAPC values of three types of tuberculosis due to smoking-related age-standardized rates for deaths and DALYs from 1990 to 2021. Abbreviations: EAPC, estimated annual percentage change; DALYs, disability-adjusted-life-years.

**Figure S6.** Three types of tuberculosis due to smoking-related GBD of deaths, YLDs, YLLs and DALYs between for different gender in 2021. Abbreviations: ASR, age-standardized rate; YLDs, Years Lived with Disability; YLLs, Years of Life Lost; DALYs, disability-adjusted-life-years.

**Figure S7.** Three types of tuberculosis due to smoking-related GBD of deaths, YLDs, YLLs and DALYs between for different age groups in 2021. Abbreviations: ASR, age-standardized rate; YLDs, Years Lived with Disability; YLLs, Years of Life Lost; DALYs, disability-adjusted-life-years.

**Figure S8.** Tuberculosis due to smoking-related GBD of deaths, YLDs, YLLs and DALYs between for different SDI regions in 2021. Abbreviations: ASR, age-standardized rate; YLDs, Years Lived with Disability; YLLs, Years of Life Lost; DALYs, disability-adjusted-life-years.

**Figure S9.** Three types of tuberculosis due to smoking-related GBD of deaths, YLDs, YLLs and DALYs between for different GBD regions in 2021. Abbreviations: ASR, age-standardized rate; YLDs, Years Lived with Disability; YLLs, Years of Life Lost; DALYs, disability-adjusted-life-years.

**Figure S10.** Changes in Deaths, DALYs, YLDs and YLLs for three types of tuberculosis due to metabolic factors according to population-level determinants of ageing, population growth, and epidemiological change from 1990 to 2021 for different SDI.

**Figure S11.** Changes in Deaths, DALYs, YLDs and YLLs for three types of tuberculosis due to metabolic factors according to population-level determinants of ageing, population growth, and epidemiological change from 1990 to 2021 for different gender.

**Figure S12**. The predicted results in three types of tuberculosis due to metabolic factors-related GBD of deaths, YLDs, YLLs and DALYs from 2022 to 2050 by ARIMA and ES model. Abbreviations: ASR, age-standardized rate; YLDs, Years Lived with Disability; YLLs, Years of Life Lost; DALYs, disability-adjusted-life-years.

**Figure S1.** Three types of tuberculosis due to smoking-related GBD of deaths, YLDs, YLLs and DALYs between 1990 and 2021. Abbreviations: ASR, age-standardized rate; YLDs, Years Lived with Disability; YLLs, Years of Life Lost; DALYs, disability-adjusted-life-years.


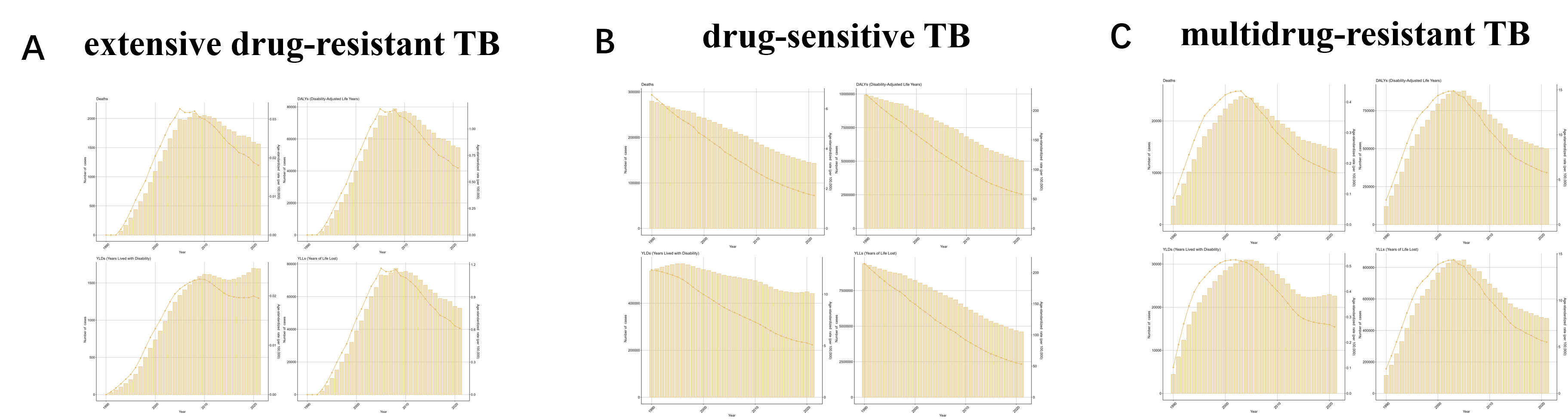


**Figure S2.** Three types of tuberculosis due to smoking-related GBD of deaths, YLDs, YLLs and DALYs between for different gender between 1990 and 2021. Abbreviations: ASR, age-standardized rate; YLDs, Years Lived with Disability; YLLs, Years of Life Lost; DALYs, disability-adjusted-life-years.


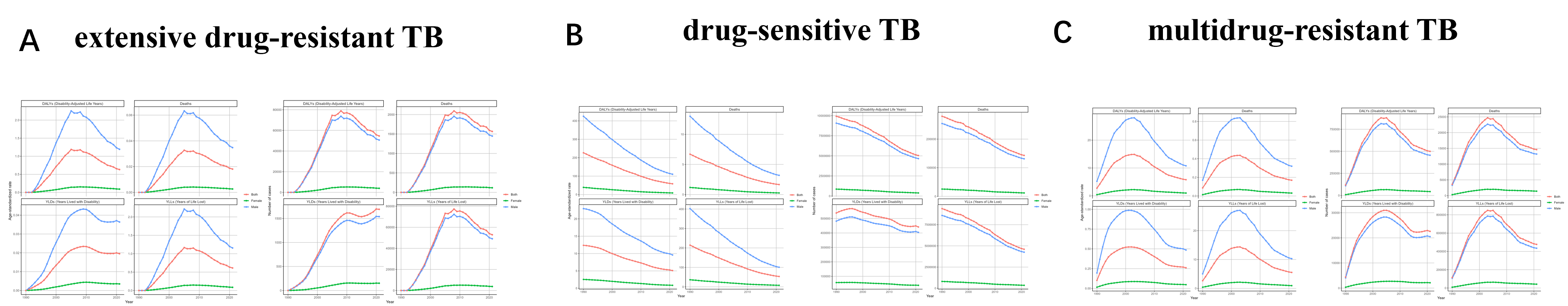


**Figure S3.** Three types of tuberculosis due to smokin-related GBD of deaths, YLDs, YLLs and DALYs between for different ages between 1990 and 2021. Abbreviations: ASR, age-standardized rate; YLDs, Years Lived with Disability; YLLs, Years of Life Lost; DALYs, disability-adjusted-life-years.


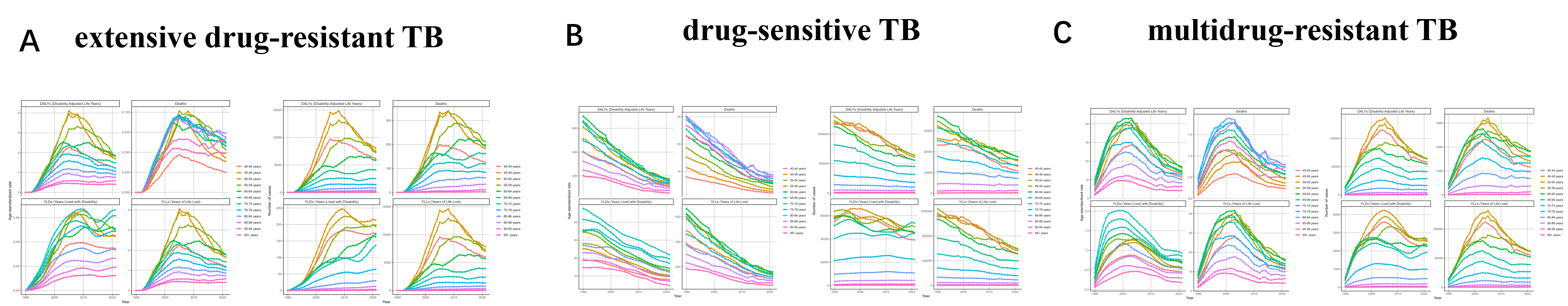


**Figure S4.** Three types of tuberculosis due to smoking-related GBD of deaths, YLDs, YLLs and DALYs between for different SDI regions between 1990 and 2021. Abbreviations: ASR, age-standardized rate; YLDs, Years Lived with Disability; YLLs, Years of Life Lost; DALYs, disability-adjusted-life-years.


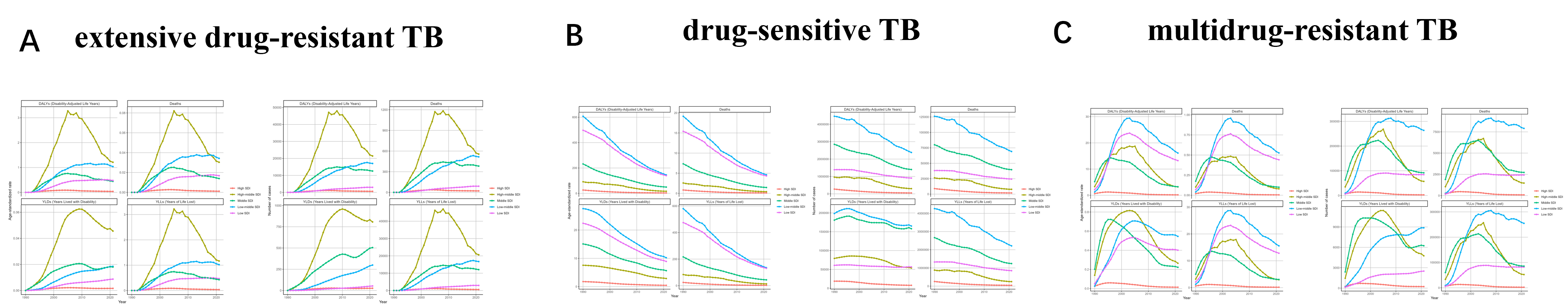


**Figure S5.** Results of cluster analysis based on the EAPC values of three types of tuberculosis due to smoking-related age-standardized rates for deaths and DALYs from 1990 to 2021. Abbreviations: EAPC, estimated annual percentage change; DALYs, disability-adjusted-life-years.


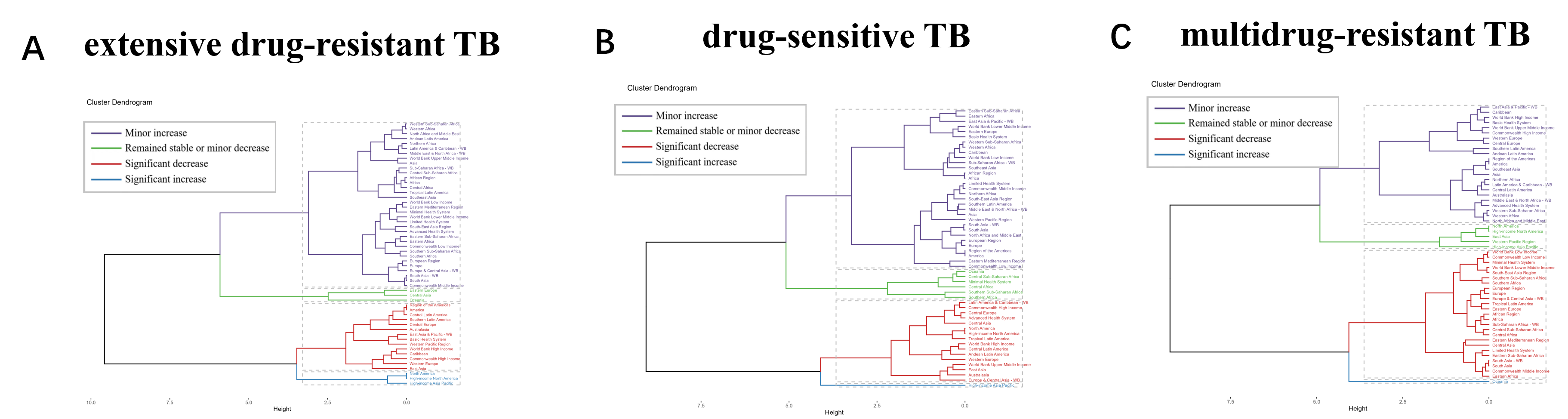


**Figure S6.** Three types of tuberculosis due to smoking-related GBD of deaths, YLDs, YLLs and DALYs between for different gender in 2021. Abbreviations: ASR, age-standardized rate; YLDs, Years Lived with Disability; YLLs, Years of Life Lost; DALYs, disability-adjusted-life-years.


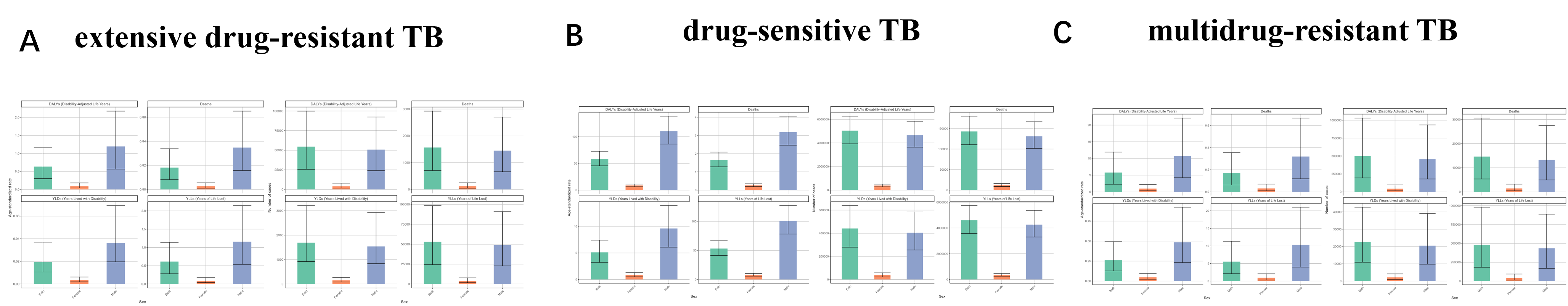


**Figure S7.** Three types of tuberculosis due to smoking-related GBD of deaths, YLDs, YLLs and DALYs between for different age groups in 2021. Abbreviations: ASR, age-standardized rate; YLDs, Years Lived with Disability; YLLs, Years of Life Lost; DALYs, disability-adjusted-life-years.


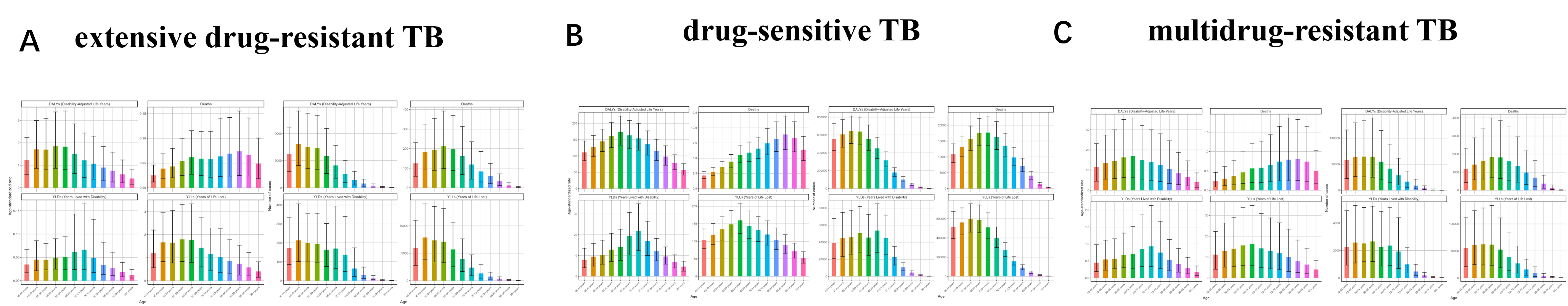


**Figure S8.** Tuberculosis due to smoking-related GBD of deaths, YLDs, YLLs and DALYs between for different SDI regions in 2021. Abbreviations: ASR, age-standardized rate; YLDs, Years Lived with Disability; YLLs, Years of Life Lost; DALYs, disability-adjusted-life-years.


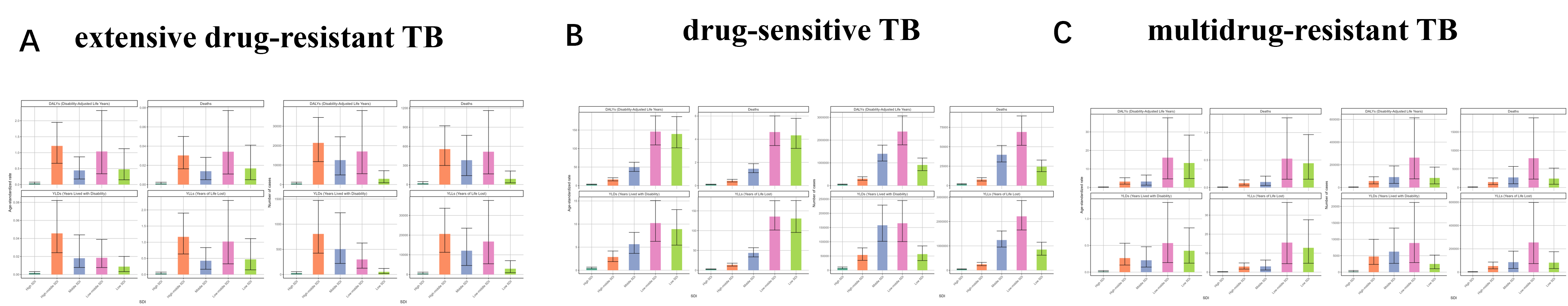


**Figure S9.** Three types of tuberculosis due to smoking-related GBD of deaths, YLDs, YLLs and DALYs between for different GBD regions in 2021. Abbreviations: ASR, age-standardized rate; YLDs, Years Lived with Disability; YLLs, Years of Life Lost; DALYs, disability-adjusted-life-years.


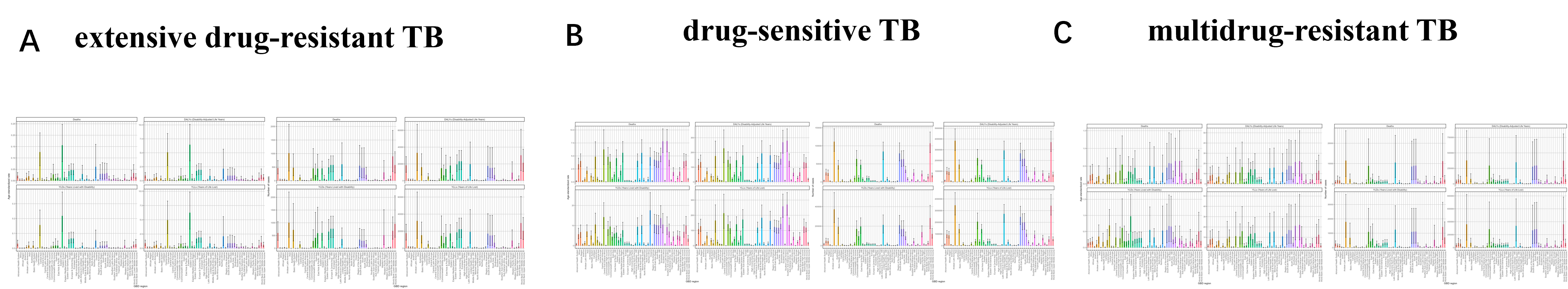


**Figure S10.** Changes in Deaths, DALYs, YLDs and YLLs for three types of tuberculosis due to metabolic factors according to population-level determinants of ageing, population growth, and epidemiological change from 1990 to 2021 for different SDI.


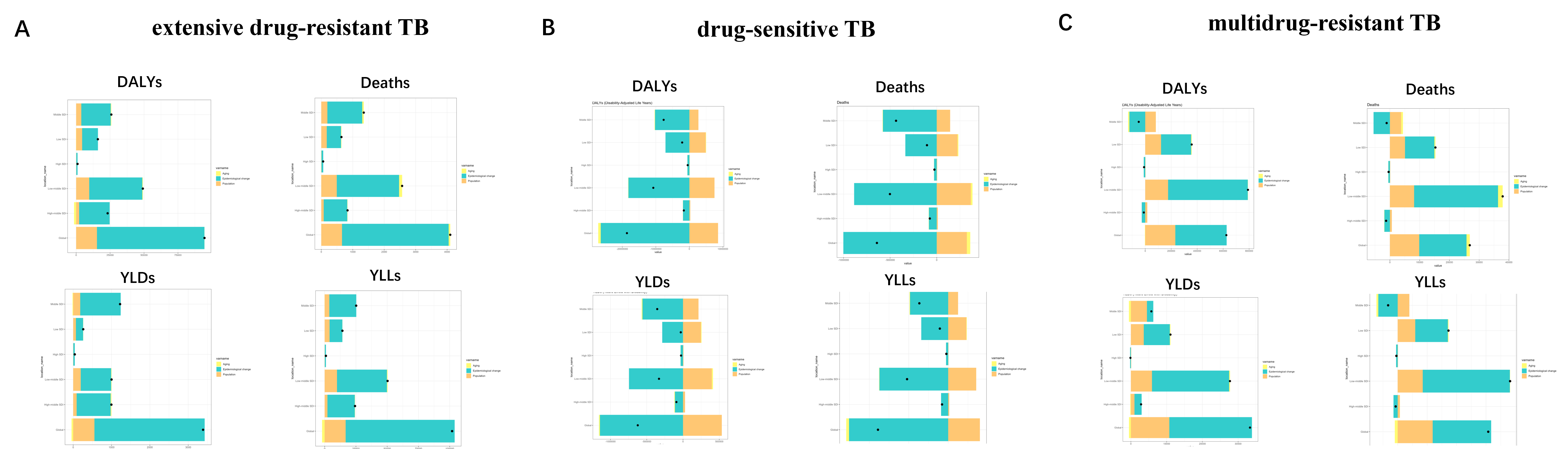


**Figure S11.** Changes in Deaths, DALYs, YLDs and YLLs for three types of tuberculosis due to metabolic factors according to population-level determinants of ageing, population growth, and epidemiological change from 1990 to 2021 for different gender.


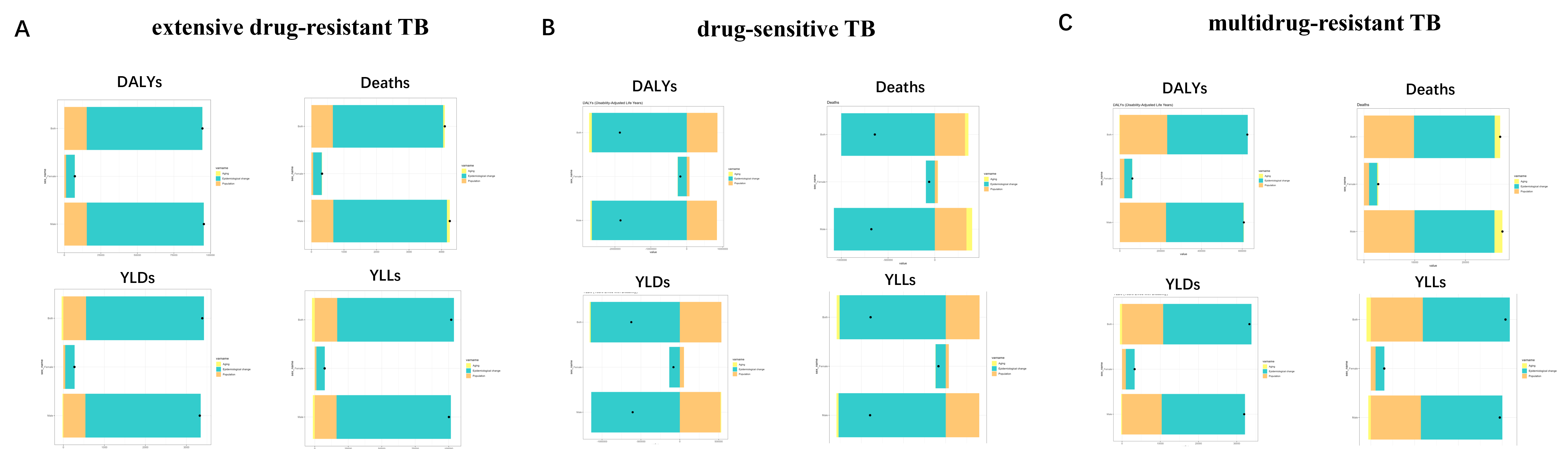


**Figure S12**. The predicted results in three types of tuberculosis due to metabolic factors-related GBD of deaths, YLDs, YLLs and DALYs from 2022 to 2050 by ARIMA and ES model. Abbreviations: ASR, age-standardized rate; YLDs, Years Lived with Disability; YLLs, Years of Life Lost; DALYs, disability-adjusted-life-years.


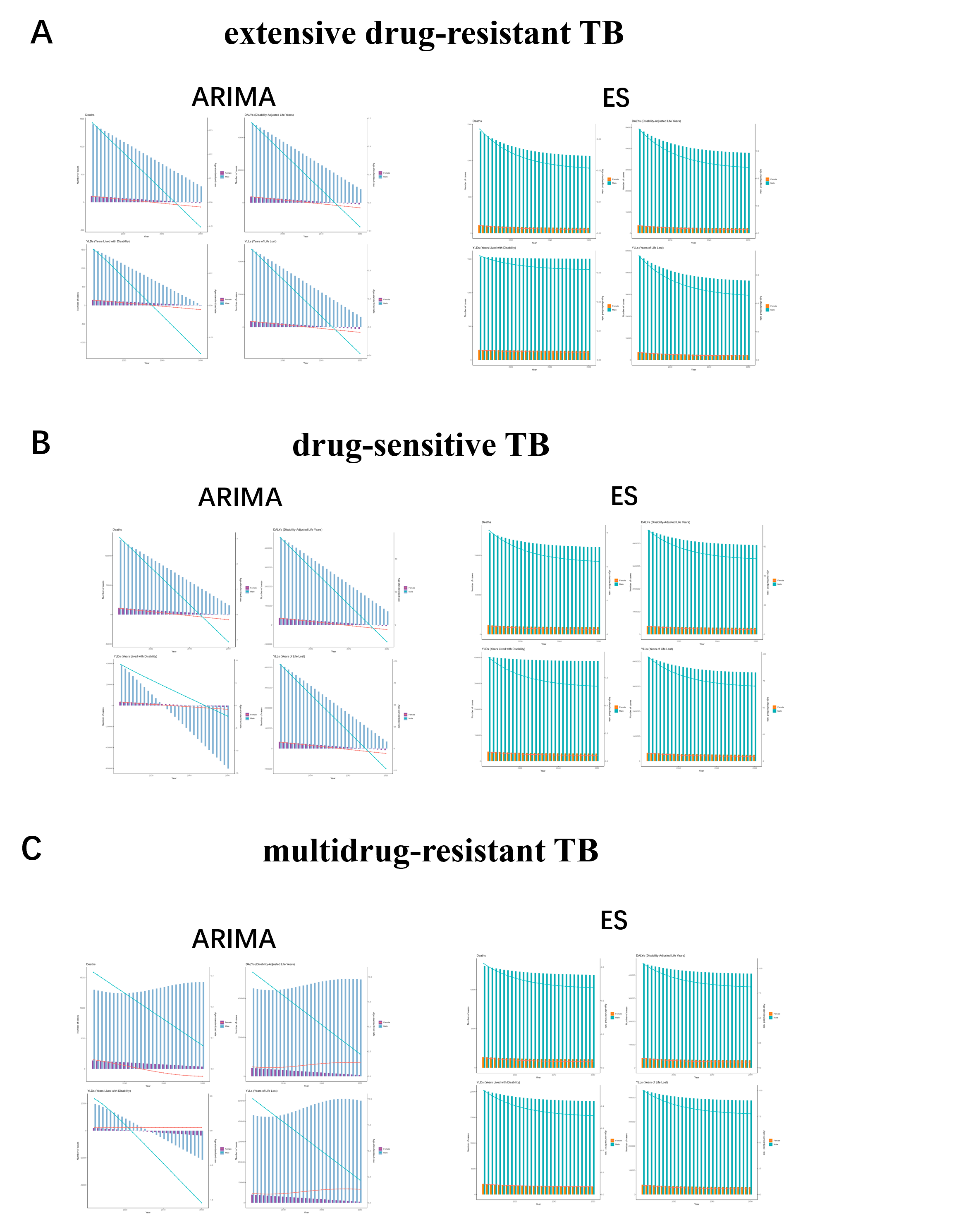

Supplement: Supplementary file 6 [file SupplementaryFile1.docx]
